# The complete chloroplast genome sequence of *Tapiscia sinensis* (Staphyleaceae)

**DOI:** 10.1080/23802359.2020.1781565

**Published:** 2020-07-06

**Authors:** Jing-Yao Zhang, De-Qiang Chen, Shuang Xiang, Xi Wu, Yi-Fan Wang, Yi-Xun Le, Wei-Hong Sun, Shuang-Quan Zou, Yun-Wei Zhou, Xiao-Xing Zou

**Affiliations:** a College of Landscape Architecture, Northeast Forestry University, Harbin, China; b College of Forestry, Fujian Agriculture and Forestry University, Fuzhou, China; c Fujian Colleges and Universities Engineering Research Institute of Conservation and Utilization of Natural Bioresources, College of Forestry, Fujian Agriculture and Forestry University, Fuzhou, China; d Key Laboratory of National Forestry and Grassland Administration for Orchid Conservation and Utilization at College of Forestry, Fujian Agriculture and Forestry University, Fuzhou, China

**Keywords:** *Tapiscia sinensis*, plastid genome, phylogeny

## Abstract

*Tapiscia sinensis*, belong to Tapisciaceae, is endangered tree endemic to China. Here, we provide the complete plastid genomic data of *T. sinensis* with the aim of providing data for future conservation efforts research and revealing its phylogenetic position. The complete chloroplast sequence is 161,093 bp, including a large single-copy (LSC) region of 87,782 bp, a small single-copy (SSC) region of 18,517 bp, a pair of invert repeats (IR) regions of 27,387 bp. Plastid genome contains 131 genes, 85 protein-coding genes, 38 tRNA genes, and eight rRNA genes. Phylogenetic analysis base on 19 plastid genomes indicates that *T. sinensis* located Malvids branch, and is more closely related to the species of the order Sapindales than those of the order Malvales.

The Tapisciaceae are part of the Huerteales, which together with Geraniales, Myrtales, Crossosomatales, Picramniales, Sapinales, Malvales, and Brassicales, constitute the malvids (APG IV 2016). *Tapiscia sinensis*, belong to Tapisciaceae, is an ancient and endangered tree endemic to China, and mainly distributed in southwestern China at an elevation of 250–2200 m (Di and Yu 1989; Zhang 1989). *Tapiscia sinensis* is a good research object to infer the refugial location and colonization history of plant species in subtropical China because it is a Tertiary relict endemic to subtropical China (Zhang, Han, et al. 2018). Wood from *T. sinensis* is used in the production of furniture due to its light wood and beautiful texture (Wei et al. 2016). The leaves of *T. sinensis* are rich in flavonoids, which are widely used in medicinal materials (Xie 2006). Besides, *T. sinensis* are often used as ornamental trees because of their straight trunks and brightly colored flowers (Zhou et al. 2015). Although *T. sinensis* was listed as a state third class protected species in China in 1992, the natural population has been fragmented and threatened due to pollination difficulties, inability to bear fruit, weak natural regeneration, and human interference (Fu 1992; Ma 2013; Cai and Zhang 2017). It is imperative to provide theoretical data for effective conservation strategies for this important plant based on molecular data. Here, we report the complete plastid genome of *T. sinensis* based on Illumina pair-end sequencing technology. This information will be helpful to study the origin, evolution, and diversification of Tapisciaceae and other species.

The fresh leaves of *T. sinensis* were collected from Fuzhou Arboretum, Fuzhou City, Fujian province, China (119°17´32′´E, 26°08´56′´N), and used for DNA extraction (Qiu et al. 2020). The voucher specimen is kept at the Herbarium of College of Forestry, Fujian Agriculture and Forestry University (specimen code FAFU08018) (Yang et al. 2019). Total genomic DNA was extracted using the modified CTAB method, and the Covaris ultrasonic breaker was used to randomly interrupted 500 bp for library construction (Xiang et al. 2019). The constructed library was sequenced PE150 by Illumina Hiseq Xten platform, approximately 2 GB data was generated (Wang et al. 2019). Illumina data was filtered by script in the cluster (default parameter: -L 5, -p 0.5, -N 0.1). The plastid genome of *T. sinensis* was assembled by GetOrganelle pipe-line (https://github.com/Kinggerm/GetOrganelle) with complete plastid genome of *Staphylea trifolia* (GeneBank accession: MK488092) as reference (Chen et al. 2019). Then, we used Bandage to view and edit these sequences of assembly (Wick et al. 2015). Assembled plastid genome annotation was conducted base on comparison with *S. trifolia* by Geneious v 11.1.5 (Biomatters Ltd., Auckland, New Zealand) (Kearse et al. 2012). The annotation result was drawn with the online tool OGDRAW (http://ogdraw.mpimp-golm.mpg.de/) (Lohse et al. 2013). The complete plastid genome sequence of *T. sinensis* has been submitted to GenBank with the accession number MT492024.

The complete plastid genome sequence of *T. sinensis* was 161,093 bp in length, with a large single-copy (LSC) region of 87,782 bp, a small single-copy (SSC) region of 18,517 bp, and a pair of inverted repeats (IR) regions of 27,387 bp. This result was basically consistent with the results reported by Zhang et al. (Zhang, Wang, et al. 2018). The complete plastid genome encodes 131 genes, containing 85 protein-coding genes, 38 transfer RNA (tRNA) genes, and eight ribosomal (rRNA) genes. The complete genome GC content was 37.2%. To reveal the phylogenetic position of *T. sinensis*, a phylogenetic tree was conducted based on complete plastid genomes of 18 species, including two Buxales species (*Pachysandra terminalis* and *Buxus microphylla*), one Proteales species (*Sabia yunnanensis*), three Fabids species (*Kandelia obovata*, *Maytenus guangxiensis*, and *Euonymus schensianus*), six Malvids species (two *T. sinensis*, *Euscaphis japonica*, *S. trifolia*, *Acer catalpifolium*, and *A. buergerianum*), four Asterids species (*Euryodendron excelsum*, *Iodes cirrhosa*, *Ilex cornuta*, and *I. asprella*), and two monocots species (*Dendrobium chrysanthum* and *D. brymerianum*) as outgroups. These genome data were downloaded from NCBI GenBank (Li et al. 2019). The sequences were aligned by MAFFT v7.307 with 1000 bootstrap replicates (Katoh and Standley 2013), and the phylogenetic tree constructed by RAxML (Stamatakis 2014). The phylogenetic tree showed that the Proteales and Buxales are located at the base of the eudicots branch, the remaining eudicots are divided into the Rosids branch and the Asterids branch. In addition, *T. sinensis* located Malvids branch, and is more closely related to the species of the order Sapindales than those of the order Malvales (Figure 1). The completed plastid genome sequence of *T. sinensis* can help reveal its phylogenetic position, and provide data for future conservation efforts and biological research.

**Figure 1.**
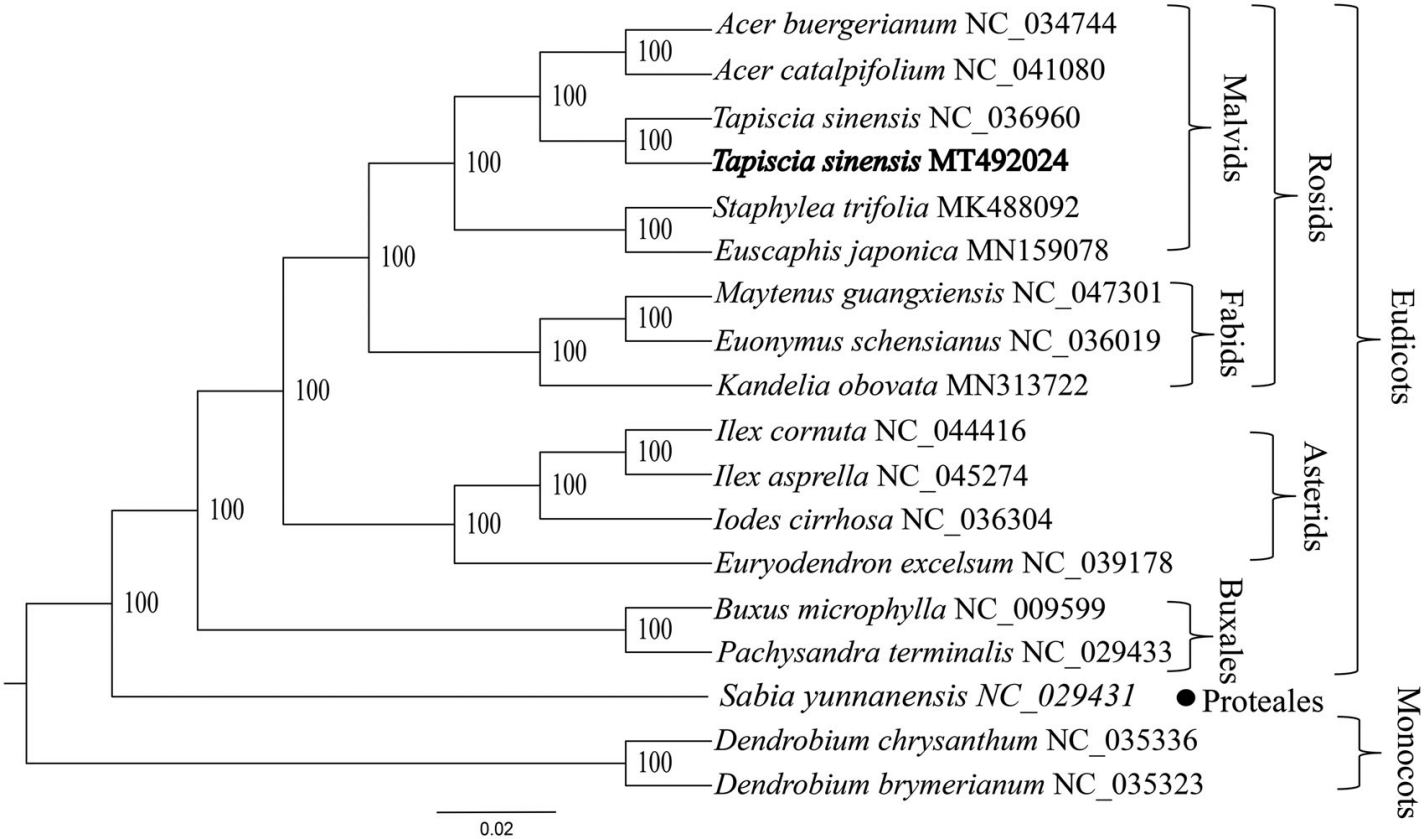
The phylogenetic tree of *Tapiscia sinensis* with other species.

## Data Availability

The data that support the findings of this study are openly available in GenBank of NCBI at https://www.ncbi.nlm.nih.gov, reference number MT492024.
